# *Rickettsia honei* Infection in Human, Nepal, 2009

**DOI:** 10.3201/eid1710.101943

**Published:** 2011-10

**Authors:** Holly Murphy, Aurélie Renvoisé, Prativa Pandey, Philippe Parola, Didier Raoult

**Affiliations:** Canadian International Water and Energy Consultants Clinic Travel Medicine Center, Kathmandu, Nepal (H. Murphy, P. Pandey);; Université de la Méditerranée, Marseille, France (A. Renvoisé, P. Parola, D. Raoult)

**Keywords:** Rickettsia honei, rickettsia, Flinders Island spotted fever, Nepal, human, ticks, dispatch

## Abstract

We report a case of *Rickettsia honei* infection in a human in Nepal. The patient had severe illness and many clinical features typical of Flinders Island spotted fever. Diagnosis was confirmed by indirect immunofluorescent assay with serum and molecular biological techniques. Flinders Island spotted fever may be an endemic rickettsiosis in Nepal.

Tick-borne rickettsioses are emerging zoonoses of marked endemicity caused by spotted fever group (SFG) rickettsia. Interest in rickettsioses is associated with increased description of new species and diseases, but this increase is unevenly distributed worldwide. Among patients with fever in Nepal, murine typhus and scrub typhus are frequently described (*1*), but tick-borne rickettsioses remain underinvestigated. *Rickettsia honei* is an SFG species that was described as a new species in 1998 and as the cause of Flinders Island spotted fever (FISF) in Australia (*2**,**3*). One human case of FISF has been confirmed in Thailand (*4*). We report a case of tick-borne rickettsiosis in Nepal caused by *R. honei* and highlight the necessity for heightened interest in emerging rickettsioses in Asia.

## The Patient

A 67-year-old woman was admitted to the Canadian International Water and Energy Consultants Clinic Travel Medicine Center in Kathmandu, Nepal, in April 2009. She had a 5-day history of fever (40.3°C), headache, diarrhea, and severe arthralgias. Results of a physical examination were unremarkable. Laboratory tests showed a leukocyte count of 6,500 cells/mm^3^, an increase in immmature neutrophils and polymorphonuclear leukocytes, and thrombocytopenia. Treatment was initiated with intravenous ceftriaxone, 2 g every 24 h for 8 days, for suspected enteric fever.

Within 48 hours, her condition worsened. The patient had photosensitivity, tinnitus, frontal headache, insomnia, confusion, cough, distress, hypotension, tachycardia, hypoxia (88% oxygenation with 2 L of O_2_), and fever (38.4°C). She was also disoriented regarding place and time and had bilateral deafness, conjunctivitis, multiple lymphadenopathies, tender hepatosplenomegaly, bilateral rales, and a purpuric rash. The rash showed a predilection for the extremities, including palms and soles (Figure). There was no eschar. Pertinent laboratory values were the following: creatinine 2 mg/dL (baseline 0.8 mg/dL), aspartate aminotransferase 105 U/L, alkaline phosphatase 765 U/L, and minimum platelet count 40,000/mm^3^. Chest radiograph showed bilateral interstitial infiltrates.

**Figure:**
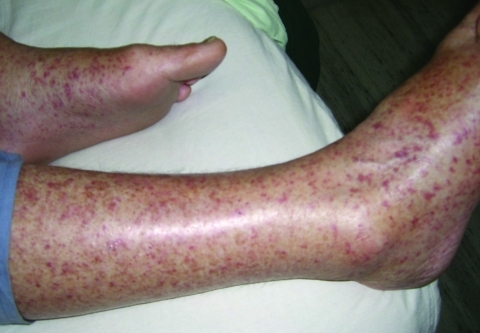
Rash exhibited by patient infected with *Rickettsia honei*, Nepal, 2009.

The patient was from New Zealand, had lived in Nepal for 30 years, and worked in wild dog protection. She reported removal of a tick 2 weeks before admission and contact with dogs, rats, ticks, fleas, and mosquitoes. She had returned from a 1-month visit to Queenstown, New Zealand, 3 months earlier and had stayed for 2 days in Thailand. She spent 1 year in Canberra, Australian Capital Territory, Australia, 12 years earlier. She was treated with oral doxycycline (100 mg 2×/d for 14 days) and showed defervescence by day 16. She recovered slowly over 3 months but had persistent tinnitus and residual high-tone hearing loss bilaterally.

Serum samples were sent to the Unité des Rickettsies (Marseille, France) to identify the etiologic agent. Samples were tested by using a multiple-antigen immunofluorescent test (*5*). Antigens included those from SFG *Rickettsia* spp., typhus group *Rickettsia* spp., and *Orientia tsutsugamushi*. Increased immunoglobulin (Ig) G and IgM titers were observed, mainly for SFG rickettsiae (Table). Kinetics of antibodies titers showed seroconversion within 3 weeks of follow-up and 4-fold increases in IgG titers, which confirm a diagnosis of rickettsial infection. As usually observed for *Rickettsia* species, serologic cross-reactivity occurred, but the highest increased antibodies titers in convalescent-phase serum were for *R. honei* (IgG 1,024, IgM 64) and *R. felis* (IgG 256, IgM 16) (Table).

**Table:** Kinetics of species-specific antibody titers in patient infected with *Rickettsia honei*, Nepal, 2009*

Species tested	IgG/IgM titer by date			
	Apr 20	Apr 24	Apr 29	May 15
*R*. *honei*	Neg	256/32	512/32	1,024/64
*R. felis*	32/0	256/16	256/16	256/16
*R. massiliae*	Neg	Neg	Neg	128/16
*R. aeschlimannii*	Neg	Neg	Neg	128/16
*R. conorii* subsp. *israelensis*	Neg	Neg	Neg	128/16
*R. conorii* subsp. *conorii*	Neg	Neg	Neg	128/16
*R. conorii* subsp. *mongolitimonae*	Neg	Neg	Neg	128/18
*R. slovaca*	Neg	Neg	Neg	128/16
*R. helijongangensis*	Neg	32/32	64/32	64/64
*R.* AT1	Neg	32/32	64/32	64/64
*R. africae*	Neg	32/32	64/32	64/64
*R. japonica*	Neg	32/32	64/32	64/64
*R. conorii* subsp. *indica*	Neg	Neg	64/32	64/32
*R. typhi*	Neg	Neg	Neg	64/64
*R. prowazekii*	Neg	Neg	Neg	64/64
*Orientia tsutsugamushi* serotype Kawasaki	Neg	Neg	Neg	64/64
*O. tsutsugamushi* serotype Gilliam	Neg	Neg	Neg	Neg

*Ig, immunoglobulin; neg, negative. Titer cutoff values were >128 for IgG and >64 for IgM. A negative titer was reported when an initial serum screening result was negative. A titer of 0 was reported when an initial screening result was positive but no Ig was detected.

The first serum sample negative for *Rickettsia* spp. was tested by real-time PCR. DNA was extracted from serum by using the QIAamp Tissue Kit (QIAGEN, Hilden, Germany), according to the manufacturer’s instructions. The result of a PCR using a probe specific for SFG *Rickettsia* spp (*6*). was positive (cycle threshold 34.3). Rickettsial DNA was detected by PCR amplifications of the outer membrane protein A (*ompA*) and *ompB* genes of *Rickettsia* spp. We obtained amplification products of 514 bp and 100% similarity with the *R. honei* *ompA* gene (GenBank accession no. AF018075) and 603 bp with 100% similarity with the *R. honei* *ompB* gene (GenBank accession nos. AF123724 and AF123711) (*2*). We obtained 2 PCR products for 2 rickettsial genes, which showed 100% sequence similarity with *R. honei*. In addition, we detected increased antibodies titers for *R. honei* antigen >2-fold higher than for any other species (Table). These results and compatible clinical features confirmed the diagnosis of *R. honei* infection.

## Conclusions

FISF was described in 1991 in Flinders Island (an island off the southeastern coast of Australia near Tasmania) and was similar to fever caused by an SFG rickettsia. In 1992, *Rickettsia honei* isolates were obtained from 2 patients with FISF. These isolates were characterized by using molecular methods and proposed as a new species in 1998 named *R. honei* (*2**,**3*); strain RB^T^ is the type strain. Thai tick typhus strain TT-118, isolated from a tick in 1962 in Thailand (*2**,**3*), was shown to be a strain of *R. honei*. *R. honei* strain marmionii was detected in 2007 (*7*); although the precise taxonomic position of this subspecies is unknown.

*R. honei* has been associated with various tick species (*3*), including *Ixodes granulatus* (Thailand) and *Haemaphysalis novaeguineae* (*7*) (mainland Australia) associated with rats, and *Aponoma hydrosauri* ticks (now *Bothriocroton hydrosauri*) (Flinders Island, Australia) associated with reptiles. One explanation for the uncommon distribution of *R. honei* is that ectoparasites associated with migrating birds that feed on local reptiles may transmit *R. honei* to reptile ticks (*3*).

Human cases of *R. honei* infection have been reported on Flinders Island and elsewhere in Australia (Tasmania, South Australia, Queensland, Torres Strait Islands) since 1991 (*8*) and in Thailand (*4*). Disease occurs primarily in spring and summer and has been mild; no deaths have been reported. Common features include fever, headache, myalgia, cough, arthralgia, and maculopapular to purpuric rash without vesiculation. An eschar is reported in 50% of cases (*2**,**3*).

Encephalitis, pneumonitis, tinnitus, and deafness in the patient are complications not reported with *R. honei* infections. Deafness has been reported with other SFG rickettsioses, particularly Rocky Mountain spotted fever (*9*) and infection with *O. tsutsugamushi* (*10*). Unsworth et al. reported 7 cases of FISF caused by *R. honei* strain marmionii that showed epidemiologic and clinical features different from those of classic FISF (*7*). Cases occurred in late summer and fall; cutaneous eschar was uncommon, and rash was not found on palms and soles of extremities (*7*). However, severe disease did not develop in any of these case-patients. The case we report differs from both patterns described. Unsworth et al. reported 1 chronic case of infection with *R. honei* strain marmionii, isolation of rickettsiae on day 27, and 1 patient with recrudescent disease (day 33) after a 10-day symptom-free period (*11*). However, data from other studies, such as detection of *R. honei* in ticks in Texas (*3*) or in blood of patients without fever (*8*), could represent PCR contamination; these results need to be confirmed (*12*).

The case in this study provides evidence for human infection with SFG rickettsiosis in Nepal, where murine typhus and scrub typhus have only recently been described (*1*) and SFG rickettsioses have only been suspected (*13*). A preliminary study reported isolation of strain TT-118 from a *Rhipicephalus haemaphysaloides* ticks in Nepal (*14*). Moreover, *Ix. granulatus* ticks, which are associated with *R. honei* in Thailand, have been found in Nepal (*15*).

We propose that FISF may be an endemic rickettsiosis in Nepal and that studies of SFG *Rickettsia* spp., particularly *R. honei* in this region, are needed. Our report of *R. honei* infection in Nepal suggests a broader geographic distribution of FISF than believed. Clinical and entomologic research may improve our understanding of the etiology of febrile illness and the neglected field of emerging rickettsioses in Asia.
